# Knowledge, Attitudes and Professional Behavior of Silver Diamine Fluoride among Dental Personnel: A Systematic Review

**DOI:** 10.3390/children9121936

**Published:** 2022-12-09

**Authors:** Intan Elliayana Mohammed, Nursharhani Shariff, Muhammad Faiz Mohd Hanim, Mohd Yusmiaidil Putera Mohd Yusof, Budi Aslinie Md Sabri, Nor Faezah Md Bohari, Annapurny Venkiteswaran

**Affiliations:** 1 Faculty of Dentistry, Universiti Teknologi MARA (UiTM), Sungai Buloh 47000, Selangor, Malaysia; 2 Ministry of Health, Putrajaya 62590, Malaysia; 3 Centre of Oral & Maxillofacial Diagnostics & Medicine Studies, Faculty of Dentistry, Universiti Teknologi MARA (UiTM), Sungai Buloh 47000, Selangor, Malaysia; 4 Institute of Pathology, Laboratory and Forensic Medicine (I-PPerForM), Universiti Teknologi MARA (UiTM), Sungai Buloh 47000, Selangor, Malaysia; 5 Centre of Population Oral Health and Clinical Prevention, Faculty of Dentistry, Universiti Teknologi MARA (UiTM), Sungai Buloh 47000, Selangor, Malaysia; 6 Centre of Paediatric Dentistry and Orthodontic Studies, Faculty of Dentistry, Universiti Teknologi MARA (UiTM), Sungai Buloh 47000, Selangor, Malaysia

**Keywords:** silver diamine fluoride, KAPs, dental personnel

## Abstract

Apart from the major drawback of black staining once lesion is arrested, few studies have indicated that dental personnel’s perception and attitude towards silver diamine fluoride (SDF) influences its usage. This report aims to provide a systematic review presenting dental personnel’s knowledge, attitudes, and perceptions, (KAPs) regarding SDF. A search of multiple electronic literature databases and a manual search were performed. The review was reported according to the Preferred Reporting Items for Systematic Reviews and Meta-Analyses 2020 statement. A comprehensive search yielded 719 publications and 14 cross-sectional studies matching the inclusion criteria. Awareness of SDF among respondents ranged from 29.6% to 87.8%, whereas awareness of its use in dentistry ranged from 14.4% to 94.5%. Regarding attitude towards SDF, the mean score ranged from 3.39 to 14.35. An increase in knowledge of and attitude towards SDF is significantly associated with the usage of SDF and may increase the usage of SDF. This review provides vital information on dental personnel’s KAPs regarding SDF. It is anticipated that the adaptation of SDF usage will be more widespread in the future, especially among children. Findings from the review may assist intervention programs to change misperceptions and to enhance awareness regarding SDF among dental personnel.

## 1. Introduction

The Global Burden of Disease Study 2017 reported that close to 3.5 billion people across the world are affected by oral diseases, with permanent teeth caries being the most prevalent condition. Globally, an estimated 2.3 billion people are suffering from permanent teeth caries and more than 538 million children are suffering from primary teeth caries [1]. Despite having a multifactorial and complex etiology, dental caries is a disease that is largely preventable by avoiding the intake of dietary sugar and through the use of preventive approaches such as fluoride in the management of the disease at both the individual and community level [2]. Moreover, prevention is simple and cost-effective, whereas treatment is not cheap, is sometimes complex, and is often very limited or not available in low- and middle-income countries. However, untreated caries still represent inequality, as not all segments of society in most countries benefit from these prevention interventions [3]. 

Therefore, a paradigm shift towards minimal-intervention dentistry that embodies a patient-centered approach to care and supports the development of evidence-based proven treatment options is needed [4], because a restorative approach alone is inadequate and unable to address oral health inequality, especially in deprived societies. This includes the use of cariostatic agents to stop the progress of the disease as part of treatment, rather than limiting the focus of treatment only to restorative options [5]. Silver diamine fluoride (SDF) is one of the cariostatic agents whose efficacy on arresting dental caries compared to other topical fluorides several systematic literature reviews have indicated [6,7]. 

Silver diamine fluoride (SDF) is a clear and high-concentration topical solution of a combination of fluoride and silver. Its efficacy in arresting caries is convincing due to the synergistic effects of silver, the antimicrobial agent of fluoride, and to promote remineralization, whereas ammonia is the solution’s concentration stabilizer [8]. SDF was first recorded as being used in Japan in 1969 and 1976. Shimizu (1976) [9] described three possible mechanisms of action of SDF’s cavity-prevention activity. First, the silver ions and inorganic compounds of SDF support the obliteration of the dentinal tubules. Secondly, the reaction between SDF and tooth-mineral components produces calcium fluoride (CaF2), which is responsible for cavity prevention, and silver phosphate (Ag3Po4) is for hardening dental caries. Third, SDF inhibits collagenase activity, hence preventing collagen degradation. Moreover, Horst et al. (2017) [10] reported that SDF-treated lesions are resistant to the growth of biofilm and the formation of more cavities because of the residue of the silver ion. This is called the “zombie effect”; the silver is reactivated when bacteria killed by silver ions are mixed with living bacteria, and the dead bacteria can effectively kill the living bacteria. A study that focused on the structural and chemical effects of SDF on the treated tooth discovered silver “microwires” that fill gaps in the lesion and pervade through adjacent dentinal tubules [11]. They hypothesized that SDF may exert its antibacterial properties through biochemical interactions and its innate capacity to integrate into the treated tooth structure. In addition, remineralization of SDF-treated lesions can be enhanced when combined with synthetic biomimetic peptides in which a calcium-phosphate nanocomposite surface that is capable of being restored with conventional adhesive dental composites is produced [12]. Several published systematic reviews confirmed the effectiveness of SDF in arresting dentin caries in primary teeth, with high success rates ranging from 65% to 91% [13,14]. 

SDF usage for managing dental caries in both primary and permanent teeth is not new in dentistry. However, it was only commonly used in the United States of America starting in 2014, when the use of SDF was cleared by the Food and Drug Administration (FDA) as an agent to treat tooth hypersensitivity and, in an off-label indication, for cavity-arrestment management [15]. It has also gained popularity in dental research and clinical work around the world in recent years [16]. Despite that, plus a growing evidence base and reported higher efficacy than other types of topical fluoride for cavity prevention and arrest, the use of SDF is still not common in dentistry and is not yet widely adopted in many countries. In the Netherlands, only 33% of dental practitioners use SDF, and among them, pediatric dentists use SDF more than general dentists, but neither use it on a daily basis [17]. In most studies, the usage of SDF has been reported but not the exact figure of the usage, or the findings from the study may not possible to generalize to the whole population of the country. The reason for this low adoption is still unclear. However, apart from the major drawback of SDF application, which is the occurrence of black staining on the tooth lesion once it is arrested, few studies have indicated that dental personnel’s perceptions and attitudes towards SDF have influence on its usage [18,19]. Furthermore, numerous systematic reviews have been conducted to assess the clinical effectiveness of SDF, but until now, there has been no comprehensive systematic review to evaluate the knowledge, attitudes, and usage by dental personnel. Hence, the aim of this report is to provide a systematic review presenting dental personnel’s knowledge, attitudes, perceptions, and clinical usage regarding SDF and the possible associations among these outcome measures.

## 2. Materials and Methods

This systematic review was conducted in accordance with the Preferred Reporting Items for Systematic Reviews and Meta-Analyses (PRISMA) 2020 statement. The protocol for this review is registered with PROSPERO (ID: CRD42021269386).

### 2.1. Eligibility Criteria

The inclusion criteria were cross-sectional studies that assessed all or any part of the knowledge of, attitudes towards, or perceptions of SDF. Studies presenting SDF perceived barriers and practices related to SDF usage were also included. Formulated based on the PICO strategy, the research question for this study is “How do knowledge of, attitudes towards, and perceptions of SDF among dental personnel influence its use in practice?” Studies other than cross-sectional studies and in which the participants were not dentists/dental specialists/undergraduate or postgraduate dental students were excluded. All English publications were accepted except for review articles, case reports, letters to the editor, expert opinions, guidelines, and meeting abstracts. 

### 2.2. Search Strategy

A detailed electronic search was performed in the following databases: EBSCO, SCOPUS, PUBMED, WOS, Google Scholar, and a manual search. In each database, the search was performed systematically and independently by the main author using searches that were built around the keywords: ((silver diamine fluoride) OR (diamine silver fluoride) OR (ammoniacal silver fluoride) OR (silver ammonia fluoride) OR (silver fluoride) OR (quaternary ammonium compounds) OR (saforide) OR (Riva Star) OR (silver nitrate + caries)) AND ((knowledge) OR (awareness) OR (attitude) OR (professional usage) OR (barrier)) AND ((dental personnel) OR (general dentist) OR (dental therapist) OR (specialist) OR (dental specialist) OR (pediatric) OR (dental professional) OR (dentist) OR (dental nurse)). A predefined search filter for review articles that was limited to English articles and the dental field was included (Appendix A; Table A1, Table A2, Table A3 and Table A4). However, no time restrictions were applied. A reference list of the retrieved papers was manually screened to identify additional potential reviews for inclusion. Additional methods performed included searching records from website and registered organizations. Regular updates were also performed to include the latest articles published on the topic of interest.

### 2.3. Study Selection and Extraction

Study selection and article extraction were conducted using the Preferred Reporting Items for Systematic Reviews and Meta-Analyses (PRISMA) checklist by three independent researchers (I.E.M., N.S.H. and M.F.M.H.) in two phases. In the first phase, titles and abstracts were screened to identify preselected studies, and those that were not eligible and duplicates were excluded. The full articles were retrieved if the titles/abstracts of the studies did not contain adequate information to support the decision for inclusion and exclusion. In the second phase, the full texts of all included studies were evaluated based on the same eligibility criteria. Any disagreements were resolved until consensus was reached. Then, the data were independently extracted from each selected study by the first two researchers using an Excel spreadsheet, and they cross-checked it to ensure consistency. Any discrepancy was solved through discussion with the presence of a third researcher (M.F.M.H.) and the procedure was repeated to overcome the difference that resulted while extracting every single study. Extracted data included authors, year, study site, sample size, population, age range, measured outcomes, SDF-related knowledge, attitude, practice/usage, perception/perceived barrier or advantage, and possible association related to SDF usage.

### 2.4. Assessment of Quality

The quality of included studies was evaluated by two independent reviewers (I.E.M. and N.S.H.) using the National Heart, Lung, and Blood Institute and the National Institutes of Health (NIH) quality-assessment tool for observational cross-sectional studies [20]. The respective tool contains 14 items and the quality of paper is rated Good if it fulfills 60–100%, Fair if 50–59%, and Poor if 0–49%. Any conflicts or disagreements that arose during the quality assessment were discussed with the third reviewer (M.F.M.H.) until consensus was reached. 

## 3. Results

### 3.1. Study Selection

**Figure 1:**
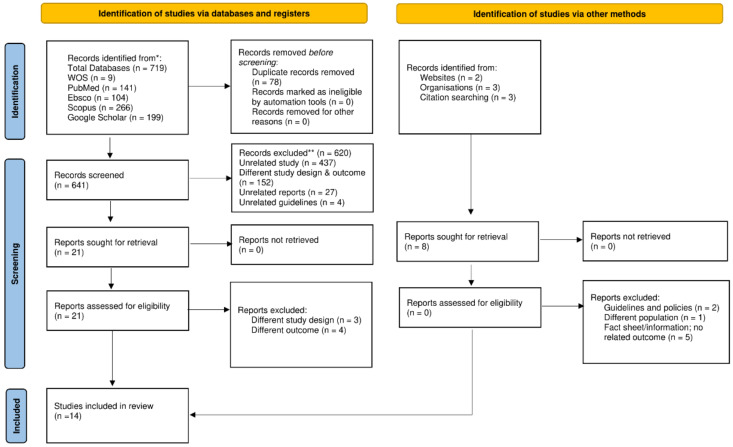
PRISMA 2020 flow diagram for the search process, which included searches of databases, registers, and other sources. * Consider, if feasible to do so, reporting the number of records identified from each database or register searched (rather than the total number across all databases/registers). ** If automation tools were used, indicate how many records were excluded by a human and how many were excluded by automation tools.

The literature search using the chosen keywords resulted in 719 citations identified through multiple sources (EBSCO, SCOPUS, PUBMED, WOS, and Google Scholar). After removal of duplicates, 641 studies were further screened based on the tittle and abstract, and 620 unrelated studies were excluded, leaving 22 studies for consideration. Identification using other methods such as websites, organizations, or citation searching was also done. However, records or studies found from these methods were not eligible to be included in the systematic review because they did not meet the exclusion and inclusion criteria. Final selection for the review consisted of 14 studies after seven studies that had no specific data needed for the review were excluded. The details of the search strategy and review of the literature identified are summarized in the PRISMA study flow chart (Figure 1). 

### 3.2. General Characteristics of Studies Included and Main Demographics of Studied Population 

Table 1 illustrates the general characteristics of the 14 included observational cross-sectional studies, which were published between 2019 and 2022. Of the studies, five (35.7%) were conducted in the United States of America [21,22,23,24,25] and another five (35.7%) were conducted in Saudi Arabia [26,27,28,29,30]. The remaining four studies were from Brazil (7.1%) [31], the Netherlands (7.1%) [17], Pakistan (7.1%) [32], and India (7.1%) [33]. Out of the 14 studies, 10 (71.4%) were performed using an online survey distributed via email or WhatsApp, two (14.3%) used both paper-based and online surveys [17,26], one (7.1%) used only a paper-based survey [24], and one (7.1%) study did not mention which method was used in distributing the questionnaire [32]. Outcomes measured from all studies included knowledge, attitude, perception/perceived barriers or advantages, SDF professional behavior or usage, and possible related associations. However, when analyzed, half of the selected studies did not categorize all variables according to the specific knowledge, attitude, or perception domains [22,23,25,26,29,30,31]. Therefore, for these studies, a discussion between two reviewers (I.E.M. and N.S.H.) was held to categorize measured variables into the indicated domains. Disagreement or conflicts regarding the issue was resolved by further discussion with the third independent reviewer (M.F.M.H.). 

Meanwhile, Table 2 demonstrates the main demographics of the studied population in the systematic review. The respondents mainly consisted of dental students (19.7%), general dentists (57.9%), and pediatric dentists (21.4%), and the respondents’ years of practice varied widely based on the studies. The sample size of included studies ranged from 58 (lowest) to 582 (highest) participants, with respondent ages from 20 to more than 60 years old. The majority of the respondents in seven studies were female (>60%) [17,26,27,28,29,32,33], in four studies the majority were male (>53%) [21,22,25,30], and three studies provided no data [23,24,31]. 

### 3.3. Quality Assessment of Selected Studies 

The quality of all selected studies in the review was assessed using the NIH quality-assessment tool (Appendix B). All studies reported the objective, study population, exposure, and outcome. However, none of them mentioned the power of the study. In 10 out of 14 studies, confounding variables were quantified and statistically adjusted using multivariate logistic regression [21,23,24,25,26,27,28,30,31,32]. To conclude, nine studies were rated good quality [21,23,24,25,27,28,30,32,33], two were rated fair quality [26,31], and the remaining three were rated poor quality. 

### 3.4. Main Outcomes 

The main outcomes of the studies included in the systematic review are summarized in Table 3. In general, most of the studies did not provide scoring (poor, moderate, good) for any domains and if scoring was provided, the system had differences that did not allow for accurate and definitive comparisons. 

#### 3.4.1. Knowledge/Awareness Related to Silver Diamine Fluoride and Related Association Factors

The majority of the respondents were aware of SDF and its use in dentistry (>60%) [22,23,24,25,28,31,32]. There was also a study that demonstrated high awareness (62.7%) when asked whether they have ever heard of SDF but at the same time were not sure or were not aware of what SDF is used for (14.9%) [30]. A study by Ezzeldin et al. (2021) [29] showed that awareness of SDF increased with the hierarchy of professional status of the respondents: students 29.6%, dentists 54.6%, and specialists 73.6% (*p*-value < 0.001).

Out of 14 included studies, three studies demonstrated low knowledge among respondents based on the percentages of knowledge-related questions answered correctly (<50%) [26,30,33]. Al Ashwal et al. (2020) [26] demonstrated that more than half of the respondents did not know anything about SDF (54.8%) and did not know the primary function of SDF (52.8%). Furthermore, the results from a study by Mehlawat et al. (2022) [33] showed that approximately only one-third of the participants had positive response regarding SDF. A total of 14.4% had good/very good knowledge about SDF use in dentistry, 13.6% had good/very good knowledge about the advantages SDF treatment can have over traditional dental treatments, 9.6% knew that SDF is used for treatment of tooth hypersensitivity, and 24.8% knew how SDF is used to treat dental caries among pediatric patients and 12.0% among adult patients. Meanwhile, only four studies performed scoring on the data using the mean, which indicated the average knowledge among the participants (mean range 1.0 to 7.4) [17,21,24,27]. There were no studies specifically indicating a high knowledge score, but studies that reported high awareness of SDF showed a high prevalence of correct answers regarding SDF, especially questions asking about patient and clinical indications (>60%) [25,28,29,31,32]. 

An increase in knowledge of SDF is significantly associated with the usage of SDF and may increase the usage of SDF. This was demonstrated in a study by Azzawi et al. (2021) included in this systematic review (*p*-value < 0.05) [28]. Apart from that, factors such as dental specialties (*p*-value < 0.001) [27], SDF professional-development education (*p*-value < 0.001) [21], and professional status (*p* < 0.001) [33] were also found to be associated with knowledge of SDF. Findings from Vollu et al. (2020) [31] showed that in regards to dental specialties, pediatric dentists had a 6.76 (95% CI [3.68, 12.41], *p*-value < 0.001) times higher chance to use SDF when compared to other dental specialties. In addition, the same study showed that dentists who work at universities had a 2.29 (95% CI [1.15, 4.57], *p*-value = 0.018) times higher chance of using SDF than those who work at private clinics. However, one study in the systematic review illustrated that no statistically significant difference was found between workplace and knowledge (*p*-value = 0.135) [30]. 

#### 3.4.2. Attitude, Professional Behavior/Usage of Silver Diamine Fluoride, and Related Associated Factors

Among the 14 studies, half did not provide data on attitude towards SDF [23,24,26,28,29,30,31]. Out of the remaining studies, four showed positive (>70%) [17,21,22,25], one demonstrated average (mean = 3.39) [27], and two showed negative (<40%) [32,33] attitudes towards SDF. In the included studies, attitude was seen mainly from the aspects of considerations of using SDF in specific clinical scenarios such as patient-related indications for SDF usage, cost-related indications for SDF usage, considerations to treatment not in aesthetic zones, and considerations to treatment in aesthetic zones [17,21,27,32,33]. In addition, in a study by Chen et al. (2019), attitude among army dentists was assessed from the considerations of using SDF based on acquired knowledge, availability, and clinical situation involving permanent teeth [22]. Questions asked consisted of “want to know more about SDF in clinical use after reading the summary of SDF” (91.38%), “interested in making SDF available in military clinics” (73.28%), “would consider using SDF if provided” (95.3%), and “would use SDF on an adult patient (permanent teeth) if it were a posterior tooth and the patient were fully aware regarding staining after caries are arrested” (87.93%).

In general, studies that showed positive attitudes showed high previous use and increased the chance of current use or future use of SDF among respondents [17,21,25]. A total of 67.0% respondents had experience using SDF to arrest carious lesions in primary teeth and 87.0% expected to increase their SDF use in the future [21]. A study in the Netherlands among dentists and pediatric dentists conducted by Schroë et al. (2022) demonstrated that current use of SDF among dentists and pediatric dentists is 16% and 74%, respectively [17]. Moreover, future use increased to 50% among dentists and remained high for pediatric dentists (74%). Meanwhile, Pizano (2017) discovered that 15% of dentists and 45% of pediatric dentists are currently using SDF in practice [25]. In a study with an average score, attitude was found to be significantly associated with dental specialties (*p* < 0.001) [27]. Low usage of SDF is in line with a study with a negative attitude toward SDF [33]. Only 10.4% used it often to treat cavity lesions in primary teeth and 21.6% expected its usage to increase significantly in the future. 

#### 3.4.3. Perceptions/Perceived Barriers or Advantages of Silver Diamine Fluoride and Related Associated Factors

SDF-related perceptions/perceived barriers or advantages were not reported in four studies in this review [21,29,32,33]. However, in regard to the other 10 studies, the majority of the participants were concerned about the permanent black staining/discoloration that may be caused by SDF after application to cavity lesions is completed (>50%) [17,23,25,26,27,28,30,31]. Apart from that, patient/parental acceptance was perceived as a significant barrier in most of the included studies as well (>30%) [17,23,27,28,31]. Furthermore, other perceived barriers included insufficient knowledge (>30%) [17,28,31] and inadequate training on SDF (>25%) [25,28]. 

A survey distributed to graduating dental students at seven dental schools in the United States indicated that the graduating students had a positive perception of SDF regarding its usefulness (*p* < 0.001) and appropriateness (*p* = 0.01), and they appeared to be more incline to utilize SDF in the future (*p* < 0.001) [24]. A study by Chhokar et al. (2017) showed that the perception of “SDF advantages outweigh the disadvantages to the patients” was statistically significant, along with “I want to offer the option of SDF so that my patients receive the best dental care” (*p*-value < 0.01). This study was carried out among dental hygienists in the United States of America. The advantages reported in the study consisted of no dental drill being needed in order to place restorative material (78%), being less expensive than restorative treatment (82%), being time efficient because of its similarity to varnish application (86%), and not requiring local anesthesia (91%) [23].

## 4. Discussion

SDF usage for managing dental caries is not new in dentistry. SDF use is recommended by the World Health Organization [34] to arrest early-childhood caries. Despite the growing evidence on its advantages and higher efficacy than other topical fluoride for cavity prevention and arrest, the use of SDF in the dental field is not yet generally adopted globally. This systematic review of KAP studies on SDF is carried out because they provide critical information for determining the appropriate intervention strategies to change misperceptions and increase understanding of SDF among dental personnel. Furthermore, it can assist program planners in evaluating their policy toward widely implementing SDF and providing suitable and adequate training in dental services, thus improving the quality of care.

In term of awareness, the majority of the studies showed high awareness of the existence of SDF and its use in dentistry. This finding is more likely a result of the respondents’ professional development, which was also found to be a factor that was significantly associated with knowledge in one of the studies included in this systematic review [21]. Increased awareness of SDF was found to be in line with the hierarchy of professional status. This is similar to a finding in another study included in this review that showed a significant association between knowledge and professional status [32]. In this case, this is because specialists may have better access to current scientific evidence, giving them knowledge and the capacity to use new products or techniques. Studies indicating low knowledge among respondents demonstrated responses that were not consistent with empirical evidence. Even though SDF is not novel in dentistry, this finding might be due to similar reasons quoted by a number of studies, which stated that it was only commonly used in the United States of America starting in 2014, when the use of SDF was cleared by the Food and Drug Administration (FDA) as an agent to treat tooth hypersensitivity and, in an off-label indication, for cavity-arrestment management [4,10]. Many dental personnel may still be lacking exposure because of limited educational experience and lack of familiarity with SDF use in everyday clinical practice. Knowledge of SDF has significant associations with the use of SDF and may lead to an increase in SDF use [28]. Vollu et al. (2020) [25] demonstrated that a 6.76 times higher chance was observed in pediatric dentists to use SDF when compared with other dental specialties. If compared with general dentistry or other dental specialties, pediatric dentists commonly have more child patients, as they provide thorough care to those who have behavioral or cooperation issues. Minimal-intervention approaches such as SDF are particularly beneficial to these children, and it is likely that pediatric dentists will use it more when delivering dental care to them [35]. Furthermore, SDF is known to be used often in treating cavity lesions in primary teeth. In addition, the American Academy of Pediatric Dentistry (AAPD) recommended SDF usage and adopted a policy and guideline supporting its use to treat caries in primary teeth as part of a comprehensive cavity-management program [36,37]. 

The attitude of dental practitioners toward preventive dentistry is a crucial element that might affect their decision to apply preventive dental treatment and their capacity to convince patients to seek preventive care [38]. This is applicable to SDF as well. Attitudes towards SDF were seen to be more in the range of average to positive in the review even though the other half of the studies did not report on its outcome. In general, these studies demonstrated positive attitude towards SDF, which contributed to the high or acceptable current use rate and possible increased use of it in the future. This is supported by a study conducted in India among dental practitioners assessing the KAPs pertaining to preventive dental care [39]. It was shown that favorable and positive attitudes were found to be associated with good utilization of sealants and topical fluoride [40]. In addition, in the review, a significant correlation was found between attitude and professional status [32]. Professional status in this context refers to consultants who works at universities. As mentioned in an early paragraph in this section, the same study demonstrated that knowledge was also significantly associated with professional status. Those who work at universities have more advantages in acquiring knowledge and training in a new and advanced material or technique. Similarly, the greater the consultant’s experience and expertise, the higher their confidence and usage of these techniques. Having said that, one could argue that acquiring more information about SDF would be sufficient to boost its usage; nevertheless, it has long been recognized that knowledge alone is insufficient to alter clinical behavior [41]. In order to enhance the usage of SDF in dental practice, it may be necessary to raise awareness, provide knowledge and training, make SDF more available, and normalize the application as a standard procedure for prevention or treatment.

A plethora of studies has shown that the only major drawback of SDF application is the occurrence of black staining on the tooth lesion once it is arrested [42]. This is consistent with the findings from most of the studies selected in the review. The discoloration caused by SDF is closely related to the next most common barrier perceived by more than half of the respondents, which is patient or parental acceptance. In a qualitative study comprising semi-structured telephone and face-to-face audio-recorded interviews that was conducted between December 2018 and June 2019 among National Health Service (NHS) dentists, the concern on the aesthetic outcomes of SDF treatment due to the permanent black staining of arrested caries lesion were explored and were reported as a potentially major barrier to parents’ acceptability of its use [18]. Crystal et al. (2020) reported similar results in a 2015 survey in which parental acceptance was still seen as one of the biggest obstacles to the usage of SDF [35]. Another study on parental acceptance indicated that parents consider SDF application more tolerable when the black staining is not clearly noticeable, and this barrier was found to be associated with parent education, income, and ethnicity [43]. Nevertheless, it can be seen that the acceptance of SDF treatment among parents and provision by dental personnel is growing. Based on several studies regarding parental acceptance of SDF’s “black stain” side effect on primary teeth, higher acceptance and tolerance was found for lesion staining on posterior teeth compared to on anterior teeth, unless their child had significant behavioral barriers or difficult management for dental treatment [43,44] In the extreme instance of having to decide between SDF application or their child having to undergo treatment under general anesthesia, parents’ acceptance rates of SDF staining increased for both posterior teeth and anterior teeth [43]. In contrast, perceived advantages reported in the review included the SDF procedure being deliverable even without powered dental equipment, being cheaper than restorative treatment, being time efficient because of its similarity to varnish application, and not requiring local anesthesia [23]. Positive perceptions of SDF (related to SDF usefulness and appropriateness) were also shown to lead to a higher possibility of SDF usage in the future [24]. This is because SDF has countless benefits and advantages to make one consider using it in individual practice or in the community. SDF treatment makes it possible to avoid conventional treatments such as restorations or dental extractions in patients who need behavioral or medical management [36]. SDF allows for a minimally invasive treatment that is actually useful for improving cooperativeness of anxious or pre-cooperative individuals [4]. Apart from that, SDF may allow access to areas that are impossible to reach with traditional approaches, including furcation, surrounding or underneath existing restorations, and partially erupted third molars [10].

This study has several limitations. All the included studies were cross-sectional studies, and it is the nature of cross-sectional studies to only allow for the determination of the association between dependent and independent variables but not the causal relationship. Furthermore, information from the participants was gathered using a self-administered questionnaire, which could potentially lead to recall and reporting bias. Another limitation is that other languages were filtered and only English-language studies were included in the review. Apart from that, the outcomes from half of the selected studies were not categorized according to the specific domains of the KAPs. Furthermore, in general, most of the studies did not provide scoring (poor, moderate, good) for any domains, and if scoring was provided, the system had differences that did not allow for accurate and definitive comparisons. Despite all of the limitations stated, to the researchers’ knowledge this is the first systematic review that assessed the KAPs on silver diamine fluoride among dental personnel. In addition, all possible dimensions of knowledge, attitude, practice, perception, and related associations were also discussed.

## 5. Conclusions

The findings from this review revealed a useful picture of KAPs regarding SDF among dental personnel. SDF provides numerous benefits to the population and may also reduce the inequalities of untreated caries between and within countries. It presents an opportunity for cost-effective outreach and community-based programs through application of primary and secondary care for prevention, arrest, and sensitivity. It could also be the interim in primary care to arrest caries until an appointment for definitive treatment, or to arrest caries until exfoliation. Moreover, the feasibility of the procedure, which does not require powered dental equipment (no aerosol-generating procedure), and its minimally invasive treatment are also beneficial during pandemics with airborne transmission, such as the COVID-19 pandemic. It is anticipated that the SDF usage will be adapted and become more widespread in the future, especially among children. In general, a high percentage of knowledge, positive attitude, and increased usage of SDF can be seen more among pediatric dental specialists. Therefore, this review can help program planners evaluate their policy toward incorporating SDF widely and providing proper and adequate training in dental schools and dental services, which should involve other dental personnel, including general dentists, dental hygienists, and dental students. In addition, it is also important to assess dental personnel’s KAPs regarding SDF because KAP studies provide vital information to help decide the best intervention programs to change misperceptions and to enhance awareness regarding SDF among dental personnel. 

## Figures and Tables

**Table 1:** Overview of general characteristic of studies included in the systematic review.

Author/Year	Country	Type of Study	Data Collection	Measured Outcome
Antonioni et al., 2019 [21]	United States of America	Cross-sectional	Web-based survey, validated questionnaire	Knowledge, attitudes, SDF usage
Chen et al., 2019 [22]	United States of America	Cross-sectional	Online survey (distributed via email, validation of questionnaire not mentioned)	Knowledge, attitudes, perceived barriers
Chhokar et al., 2017 [23]	United States of America	Cross-sectional	Online survey distributed via email, pre-test carried out with 12 dental personnel (dental hygienists and dentists)	Knowledge, perceptions, SDF usage
Dang et al., 2020 [24]	United States of America	Cross-sectional	Paper distribution, content validation	Knowledge, perceptions, SDF usage
Pizano, 2017 [25]	United States of America	Cross-sectional	Online survey distributed via email, questions developed by a group of experts and pilot tested	Knowledge, attitudes, perceptions, barriers, SDF usage
Al Ashwal et al., 2020 [26]	Saudi Arabia	Cross-sectional	Papers and online survey, validation of questionnaire not mentioned	Knowledge, perceived barriers to SDF, professional usage
Alajlan et al., 2020 [27]	Saudi Arabia	Cross-sectional	Online survey, validated and published questionnaire	Knowledge, attitudes, perceived barriers, SDF usage
Azzawi et al., 2021 [28]	Saudi Arabia	Cross-sectional	Online survey (Google Form), pretested questionnaire	Knowledge, perceive barriers, SDF usage
Ezzeldin et al., 2021 [29]	Saudi Arabia	Cross-sectional	Online survey, distributed via WhatsApp message, validation of questionnaire not mentioned	Knowledge/awareness, SDF usage
Alshammari et al., 2021 [30]	Saudi Arabia	Cross-sectional	Online survey using a pretested questionnaire	Knowledge, perceived barriers, SDF usage
Vollu et al., 2020 [31]	Brazil	Cross-sectional	Online survey distributed via email, validation of questionnaire not mentioned	Knowledge, perceived barriers, SDF usage
Schroë et al., 2022 [17]	The Netherlands	Cross-sectional	Paper questionnaire or online survey, pretested	Knowledge, attitudes, SDF usage
Abbas et al., 2021 [32]	Pakistan	Cross-sectional	Survey (not reported on paper or online), pretested questionnaire	Knowledge, attitudes
Mehlawat et al., 2022 [33]	India	Cross-sectional	Online survey distributed via WhatsApp group, pretested questionnaire	Knowledge, attitudes, SDF usage

**Table 2:** Overview of main demographic characteristics of studied populations.

Author/Year	Study Population	Sample Size and Response Rate (%)	Gender	Age	Years in Practice (Practitioner/Student)
Antonioni et al., 2019 [21]	Pediatric dentists	582 (9.34%)	Male 53% Female 47%	Mean age 45.68 ± 12.71 years (range 26–79 years)	Graduation year Degree 1997 ± 13.08 years (range 1964–2015) Specialist 2001 ± 12.92 years (range 1968–2016)
Chen et al., 2019 [22]	Army dentists (general dentists, comprehensive dentists, pediatric dentists)	237 (31.73%)	Male 70.89% Female 28.27%	NR	<2 years 26.16% 2–5 years 37.97% 6–9 years 15.19% 10–13 years 8.02% 14–17 years 4.22% ≥18 years 8.44%
Chhokar et al., 2017 [23]	Dental hygienists	103 (46.0%)	NR	NR	<1 year 12.0% 1–3 years 27.0% 4–6 years 21.0% 7–10 years 26.0% >10 years 14.0%
Dang et al., 2020 [24]	Dental students	386 (55.0%)	NR	Median age 27 ± 4.2 years (range 23–47 years	Graduating dental students
Pizano, 2017 [25]	Dentists, pediatric dentists	455 (11.3%)	Pediatric dentists Male 60.0% Female 40.0% Dentists Male 65.0% Female 35.0% Pediatrician Male 15.0% Female 85.0%	Pediatric dentists 20–40 years 50.0% 41–60 years 31.0% ≥61 years 19.0% Dentists 20–40 years 37.0% 41–60 years 37.0% >61 years 26.0%	Pediatric dentists 1–10 years 45.0% 11–20 years 17.0% >20 years 38.0% Dentists 1–10 years 33.0% 11–20 years 15.0% >20 years 52.0%
Al Ashwal et al., 2020 [26]	Dental students and interns	252 (Response rate was not mentioned)	Male 39.7% Female 60.3%	≤25 years 79.4% >25 years 20.6%	Level 11 33.7% Level 12 42.9% Intern 23.4%
Alajlan et al., 2020 [27]	Dental practitioners in public hospitals	278 (100%)	Male 39.93% Female 60.07%	24– 35 years 75.0% 36–45 years 17.27% >45 years 7.55%	NR
Azzawi et al., 2021 [28]	Pediatric dentists	58 (58.0%)	Male 27.6% Female 72.4%	25–40 years 62.4% >40 years 27.6%	Not stated
Ezzeldin et al., 2021 [29]	Dental students, dentists, and specialists	312 (22.8%)	Students Male 30.6% Female 69.4% Dentists Male 33.8% Female 66.2% Specialists Male 43.1% Female 56.9%	Students 20–25 years 89.9% 25–45 years 10.1% Dentists 20–35 years 90.9% 36–45 years 7.7% >50 years 1.4% Specialists 20–35 years 54.2% 36–45 years 27.7% >50 years 18.1%	Students ≤1 year 100.0%% Dentists ≤1 year 43.2% 2–5 years 24.5% 6–10 years 24.5% 11–20 years 5.1% >20 years 2.9% Specialists ≤1 year 9.9% 2–5 years 23.9% 6–10 years 18.3% 11–20 years 29.6% >20 years 18.3%
Alshammari et al., 2021 [30]	Dentists	150 (83.3%)	Male 62.0% Female 38.0%	25–35 years 98.7% 35–45 years 1.3%	Not stated
Vollu et al., 2020 [31]	Dentists	409 (3.89%)	NR	NR	Mean 16.83 ± 11.64 years
Schroë et al., 2022 [17]	Dentists, pediatric dentists	167 (25.0%)	Dentists Male 46.0% Female 54.0% Pediatric dentists Male 15.0% Female 85.0%	Dentists <35 years 41.0% 35–50 years 25.0% >51 years 34.0% Pediatric dentists <35 years 4.0% 35–50 years 70.0% >51 years 26.0%	Dentists <1 year 1.0% 1–5 years 30.0% 6–10 years 11.0% >10 years 58.0% Pediatric dentists 1–5 years 4.0% 6–10 years 4.0% >10 years 92.0%
Abbas et al., 2021 [32]	Dentists	223 (Response rate not mentioned)	Male 21.2% Female 78.8%	20–30 years 63.2% 31–40 years 30.0% 41–50 years 6.7%	NR
Mehlawat et al., 2022 [33]	Dentists	127 (63.5%)	Male 24.8% Female 75.2%	NR	NR

NR = not reported.

**Table 3:** Overview of main outcomes in the selected studies.

Author/Year	Knowledge/Awareness	Attitude/Consideration	Perception/Perceived Barrier or Advantage	Practice/Professional Usage	Associated Factor
Antonioni et al., 2019 [21]	• General SDF knowledge Mean 3.63 • Cavitated-lesion knowledge Mean 4.11 • Non-cavitated-lesion knowledge Mean 3.88 • SDF use prior to all restorative treatment Mean 1.94 (Range 1.00–5.00)	• Patient-related indications for SDF usage Mean 4.24 • Cost-related indications for SDF usage Mean 3.74 • Considerations to treatment not in aesthetic zone Mean 4.07 • Considerations to treatment in aesthetic zone Mean 2.66 (Range 1.00–5.00)	NR	Office use of SDF Mean 2.46 (Range 1.00–5.00) • 67.0% used SDF to arrest carious lesions in primary teeth • 87.0% expected to increase their SDF use in the future	Knowledge ↑ • SDF professional development education SDF usage ↑ • Positive SDF related attitude SDF usage ↓ • Older age
Chen et al., 2019 [22]	• Had heard of SDF 87.76% • Knows that SDF arrests caries in the dentin 94.51% • Knows how to apply SDF 47.68%	• Want to know more about SDF in clinical use after reading the summary of SDF 91.38% • Interested in making SDF available in military clinics 73.28% • Would consider using SDF if provided 95.3% • Would use SDF on an adult patient (permanent teeth) if it was a posterior tooth and the patient was fully aware regarding the staining after caries are arrested 87.93%	• SDF may weaken bonding strength for resin restorations 2.59% • Prefer to use other materials for cavity prevention and remineralization 8.19% • SDF is a technique-sensitive procedure that is not simple to apply 3.02% • Do not believe in the effectiveness of SDF 2.16%	NR	NR
Chhokar et al., 2017 [23]	• Had never heard of SDF 32% • Had heard of SDF but not sure what it is used for 22% • Aware of what SDF is used for 43% • Had observed SDF being used 1%	NR	• Alternative treatment to removing tooth structure with a dental drill 82% • Less expensive 82% • Applied like a varnish and therefore is time efficient 86% • Does not require the use of LA 91% • Reduced acceptance from parents/patients due to the permanent black staining 56% • SDF advantages > disadvantages to the patients I am accustomed to treating as a dental hygienist 95%	• Used SDF once 0% • Used SDF a few times 0% • Use SDF occasionally 1% • Use SDF frequently 1%	SDF advantages > disadvantages • Intention to offer SDF to patient Perception
Dang et al., 2020 [24]	• SDF properties Average score 4 average Range 0–6 • SDF indication Average score 1 average Range 0–2	NR	• SDF usefulness Median score 3 (SD = 1.25, range: −4–4) • SDF appropriateness Median score 2 (SD = 2.48, range: −6–6) • Students’ willingness to use SDF Median score 1 (SD = 0.74, range: −2–2), • Patients’ willingness to use SDF Median score 1 (SD = 0.86, range: −2–2)	Clinical experiences using SDF for older adults 54.8%	“SDF usefulness,” “SDF appropriateness,” and “SDF patient willingness to use” were significantly associated with higher perception scores of student willingness to use SDF
Pizano JM, 2017 [25]	Patients indicated for SDF treatment: • Uncooperative 0–3 years old patients Pediatric dentists 76% Dentists 73% • Patients with special health care needs Pediatric dentists 71% Dentists 73%	• Appropriate for treatment on primary posterior teeth Pediatric dentists 81% Dentists 94%	• Pediatric dentists Aesthetics 83% Not covered by insurance 55% Multiple appointments are needed 38% • Dentists Aesthetics 86% Lack of training 54% Not covered by insurance 46%	• Currently using SDF in practice Pediatric dentists 45% Dentists 15%	• Willingness to provide SDF Fewer years in practice, younger age
Al Ashwal et al., 2020 [26]	• 54.8% did not know anything about SDF • 52.8% did not know the primary function • Knowledge among students and interns was low; 57.9% had little awareness of SDF	NR	• Stains 50.4% • Metallic taste 51.2%	67% did not know SDF application technique	Knowledge • Reading about SDF • Knowing SDF’s primary function
Alajlan et al., 2020 [27]	Average level with mean of 3.12 (out of 5)	Average level with mean of 3.39 (out of 5)	Mean of 4.37 • Permanent black discoloration on the tooth after treatment 55.39% • SDF treatment does not restore natural tooth anatomy and function if it is not followed by a restoration 51.08% • Concern over patient’s satisfaction with SDF treatment 37.41%	• 61.87% had never used SDF to prevent caries • 60.43% had never used SDF to arrest dental caries in primary and 61.51% in permanent teeth. • 34.53% expected that SDF usage would increase a little in the future	Knowledge • Dental specialties Attitude • Dental specialties • Clinical titles
Azzawi et al., 2021 [28]	• Knowledge regarding SDF use in dentistry > 60%. • Knowledge of general indications of SDF * Medically compromised children 74% * Behavioral issues and anxiety 73% • Knowledge of specific indications and practices of SDF * Cavitated lesions 65% * Non-aesthetic zone of primary teeth 62%	NR	Barriers: • Not enough knowledge 31% • Not well trained in its use 27% • Aesthetic is poor 28% • Patient satisfaction is lower 35% • Insufficient amount of evidence 22% • Insurance does not cover SDF 25% • SDF is not readily available commercially 49%	Had used/currently use SDF in clinical practice 56.9%	Usage of SDF ↑ • Knowledge on how to use SDF
Ezzeldin et al., 2021 [29]	• Awareness of SDF Students 29.6% Dentists 54.6% Specialists 73.6% • Efficacy of SDF Significant proportion of specialists and graduates described: SDF application to be trouble-free, cost effective, reduces the need of GA/sedation to treat pediatric patients, application does not require LA or drilling • Cosmetic outcome of SDF Significant proportion of specialists agreed: Parents’ acceptance of the tooth discoloration Parents non-acceptance of SDF usage on anterior teeth	NR	NR	Use SDF in practice Students 8.6% Dentists 8.8% Specialists 25.0%	NR
Alshammari et al., 2021 [30]	Had heard of SDF 62.7% Dentists who answered correctly for all knowledge-based questions 14.89%	NR	Advantages of using SDF • Arresting caries 55.3% SDF disadvantages • Staining the tooth 47.9% • Irritation of pulp 27.7% • Low pH 14.9%	Future implementation of SDF replacing traditional methods 72.0%	No statistically significant difference with knowledge • Gender • Age • Work sector
Vollu et al., 2020 [31]	• Had not heard of/did not know about SDF 19.7% • Indication for SDF: Non-compliant patients 75.9% Care in places with no infrastructure 68.5% Patients with no capacity for collaboration 66.7% Primary dentition 75.9% Anterior and posterior teeth 59.3% Posterior teeth 35.2%	NR	Reported barriers among: • Those that did not use SDF Lack of scientific knowledge 58.3% Tooth staining 27.6% • Those who used SDF tooth staining 90.7% Parental acceptance 64.8%	Currently using SDF 13.2%	• Dental specialties Pediatric dentists, showing a 6.76 times higher chance of using SDF than other specialties • Workplace Dentists working at universities had a 2.29 times higher chance of using SDF than those working at private offices
Schroë et al., 2022 [17]	Mean overall knowledge items (tooth indication, patient indication, mode of action, toxicity) • Dentists 6.7 • Pediatric dentists 7.4 (out of 15)	Mean score for overall attitude of the respondents • Dentists 14.35 • Pediatric dentists 16.65	• SDF users Parental acceptance, not knowing the billing code, and the risk of staining clothing/ surfaces • SDF non-users Inadequate knowledge of SDF, parental acceptance, and not knowing the legislation around SDF	Dentists • Current use 16.0% • Future use 50.0% Pediatric dentist • Current use 74% • Future use 74%	NR
Abbas et al., 2021 [32]	• Aware of SDF use in dentistry 76.2% • Aware of SDF use in tooth-hypersensitivity treatment 79.3% • Aware of SDF use for the treatment of pediatric dental caries 92.8% • Aware of SDF use in adult dental caries 68.6% • Aware of the advantages of SDF treatment over traditional dental treatment 29.1% • Aware of potential problems associated with the use of SDF 26.5%	• A better alternative treatment for children with behavioral issues 28.7% • A better treatment option for medically fragile patients 33.2% • Alternative treatment for patients with dental anxiety 35.4% • A good treatment option for patients who have recently received chemotherapy or radiation therapy 36.8% • Can be used in patients on bisphosphate treatment 38.6% • Can be used in patients requiring GA • Can be used in patients with microstomia 20.6% • Good treatment option for primary teeth not in the aesthetic zone 59.6%	NR	NR	Professional status (consultant) was found to be significantly associated with knowledge and attitude regarding SDF
Mehlawat et al., 2022 [33]	• Had good/very good knowledge of SDF use in dentistry 14.4% • Had good/very good knowledge of the advantages SDF treatment can have over traditional dental treatments 13.6% • Knew SDF is used for treatment of tooth hypersensitivity 9.6% • Knew how SDF is used to treat dental caries among pediatric 24.8% and adult patients 12.0%	• Infected soft dentin must be removed before applying SDF 34.4% • A good treatment for arresting caries when it is not possible to restore all lesions in one appointment 34.4% • Can be used to arrest cavitated lesions in enamel 58.4%, dentin 46.4%, and root-cavity lesions 31.2% • A good treatment alternative among the children with behavioral issues 40.8%, medically fragile patients 41.6%, patients with severe dental anxiety 36%, and patients undergoing or who have recently undergone radiation therapy or chemotherapy 32%	NR	SDF usage in the future Increase a lot 21.6% Increase a little 53.6% Primary teeth • Had never used SDF for treating dental caries 34.4% • Used it often in their offices 10.4% Permanent teeth • Had never used SDF 38.4% • Used it often in their offices 8%	NR

NR = not reported.

## Data Availability

Not applicable.

## Appendix A

Search Strategy.

**Table A1:** PubMed.

No.	Concept	Search String	Number
#1	Silver diamine fluoride	((((((((silver diamine fluoride) OR (diamine silver fluoride)) OR (ammoniacal silver fluoride)) OR (silver ammonia fluoride)) OR (silver fluoride)) OR (quaternary ammonium compounds)) OR (saforide)) OR (Riva Star)) OR (silver nitrate + caries)	115,098
#2	Knowledge Awareness Attitude Professional Usage Barrier	((((knowledge) OR (awareness)) OR (attitude)) OR (professional usage)) OR (barrier)	1,840,208
#3	Dental personnel	((((((((dental personnel) OR (general dentist)) OR (dental therapist)) OR (specialist)) OR (dental specialist)) OR (pediatric)) OR (dental professional)) OR (dentist)) OR (dental nurse)	1,254,895
#4	Limited to English	#1 AND #2 AND #3	141

**Table A2:** SCOPUS.

No.	Concept	Search String	Number
#1	Silver diamine fluoride	((((((((silver diamine fluoride) OR (diamine silver fluoride)) OR (ammoniacal silver fluoride)) OR (silver ammonia fluoride)) OR (silver fluoride)) OR (quaternary ammonium compounds)) OR (saforide)) OR (Riva Star)) OR (silver nitrate + caries)	134,085
#2	Knowledge Awareness Attitude Professional Usage Barrier	((((knowledge) OR (awareness)) OR (attitude)) OR (professional usage)) OR (barrier)	8,913,542
#3	Dental personnel	((((((((dental personnel) OR (general dentist)) OR (dental therapist)) OR (specialist)) OR (dental specialist)) OR (pediatric)) OR (dental professional)) OR (dentist)) OR (dental nurse)	3,535,596
#4	Limited to English	#1 AND #2 AND #3	1324
#5	Limited to English and dentistry	#1 AND #2 AND #3	266

**Table A3:** EBSCO.

No.	Concept	Search String	Number
#1	Silver diamine fluoride	((((((((silver diamine fluoride) OR (diamine silver fluoride)) OR (ammoniacal silver fluoride)) OR (silver ammonia fluoride)) OR (silver fluoride)) OR (quaternary ammonium compounds)) OR (saforide)) OR (Riva Star)) OR (silver nitrate + caries)	44,072
#2	Knowledge Awareness Attitude Professional Usage Barrier	((((knowledge) OR (awareness)) OR (attitude)) OR (professional usage)) OR (barrier)	3,283,655
#3	Dental personnel	((((((((dental personnel) OR (general dentist)) OR (dental therapist)) OR (specialist)) OR (dental specialist)) OR (pediatric)) OR (dental professional)) OR (dentist)) OR (dental nurse)	820,839
#4	Limited to English	#1 AND #2 AND #3	1055
#5	Limited to English and dentistry	#1 AND #2 AND #3	104

**Table A4:** WOS.

No.	Concept	Search String	Number
#1	Silver diamine fluoride	((((((((silver diamine fluoride) OR (diamine silver fluoride)) OR (ammoniacal silver fluoride)) OR (silver ammonia fluoride)) OR (silver fluoride)) OR (quaternary ammonium compounds)) OR (saforide)) OR (Riva Star)) OR (silver nitrate + caries)	7900
#2	Knowledge Awareness Attitude Professional Usage Barrier	((((knowledge) OR (awareness)) OR (attitude)) OR (professional usage)) OR (barrier)	3,200,272
#3	Dental personnel	((((((((dental personnel) OR (general dentist)) OR (dental therapist)) OR (specialist)) OR (dental specialist)) OR (pediatric)) OR (dental professional)) OR (dentist)) OR (dental nurse)	488,974
#4	Limited to English	#1 AND #2 AND #3	13
#5	Limited to English and dentistry	#1 AND #2 AND #3	9

1. GOOGLE SCHOLAR

Advanced search = 199

TOTAL = 719

2. Other sources

Website: https://www.rdhmag.com/patient-care/fluoride/silver-diamine-fluoride (accessed on 4 July 2022); https://www.nytimes.com/2016/07/12/health/silver-diamine-fluoride-dentist-cavities.html (accessed on 4 July 2022).

Organizations: https://www.ada.org/en/publications/ada-professional-product-review-ppr/current-issue (accessed on 4 July 2022); https://www.aappublications.org/news/2016/08/05/SilverDiamine080516 (accessed on 4 July 2022).; https://www.aapd.org/media/policies_guidelines/g_sdf.pdf (accessed on 4 July 2022).

Reference mining

Nelson et al., 2016 [45]

American Academy of Pediatric Dentistry, 2017 [46]

Association of State and Territorial Dental Directors (ASTDD), 2017 [47].

## Appendix B

**Table A5:** Assessment of Quality for Included Studies.

Study NIH Checklist for	Antonioni et al., 2019 [21]	Chen et al., 2019 [22]	Chhokar et al., 2017 [23]	Dang et al., 2020 [24]	Pizano JM, 2017 [25]	Al Ashwal et al., 2020 [26]	Alajlan et al., 2020 [27]	Azzawi et al., 2021 [28]	Ezzeldin et al., 2021 [29]	Alshammari et al., 2021 [30]	Vollu et al., 2020 [31]	Schroë et al., 2022 [17]	Abbas et al., 2021 [32]	Mehlawat et al., 2022 [33]
1. Was the research question or objective in this paper clearly stated?	√	√	√	√	√	√	√	√	√	√	√	√	√	√
2. Was the study population clearly specified and defined?	√	√	√	√	√	√	√	√	√	√	√	√	√	√
3. Was the participation rate of eligible persons at least 50%?	X	X	X	√	X	NR	√	√	X	√	X	X	NR	√
4. Were all the subjects selected or recruited from the same or similar populations (including the same time period)? Were inclusion and exclusion criteria for being in the study prespecified and applied uniformly to all participants?	√	√	√	√	√	√	√	√	√	√	√	√	√	√
5. Was a sample size justification, a power description, or variance and effect estimates provided?	X	X	X	X	X	X	X	X	X	X	X	X	X	X
6. For the analyses in this paper, were the exposure(s) of interest measured prior to the outcome(s) being measured?	X	X	X	X	X	X	X	X	X	X	X	X	X	X
7. Was the timeframe sufficient so that one could reasonably expect to see an association between exposure and outcome if it existed?	X	X	X	X	X	X	X	X	X	X	X	X	X	X
8. For exposures that can vary in amount or level, did the study examine different levels of the exposure as related to the outcome (e.g., categories of exposure, or exposure measured as a continuous variable)?	NA	NA	NA	NA	NA	NA	NA	NA	NA	NA	NA	NA	NA	NA
9. Were the exposure measures (independent variables) clearly defined, valid, reliable, and implemented consistently across all study participants?	√	NR	√	√	√	NR	√	√	NR	√	NR	√	√	√
10. Was the exposure(s) assessed more than once over time?	NA	NA	NA	NA	NA	NA	NA	NA	NA	NA	NA	NA	NA	NA
11. Were the outcome measures (dependent variables) clearly defined, valid, reliable, and implemented consistently across all study participants?	√	√	√	√	√	√	√	√	√	√	√	√	√	√
12. Were the outcome assessors blinded to the exposure status of participants?	NA	NA	NA	NA	NA	NA	NA	NA	NA	NA	NA	NA	NA	NA
13. Was loss to follow-up after baseline 20% or less?	NA	NA	NA	NA	NA	NA	NA	NA	NA	NA	NA	NA	NA	NA
14. Were key potential confounding variables measured and adjusted statistically for their impact on the relationship between exposure(s) and outcome(s)?	√	X	√	√	√	√	√	√	X	√	√	X	√	X
CD = cannot determine; NA = not applicable; NR = not reported.														
√, Yes; X, No.														
